# Novel operative technique of advancement urethral meatoplasty utilizing buccal mucosa for Vulvar Paget’s disease with urethral invasion: two case reports

**DOI:** 10.1186/s13256-021-02729-1

**Published:** 2021-03-28

**Authors:** Masaki Nakamura, Yuji Hakozaki, Shohei Iwata, Yusuke Sato, Katsuhiro Makino, Taketo Kawai, Yuta Yamada, Daisuke Yamada, Motofumi Suzuki, Jun Omatsu, Masanobu Abe, Kazuto Hoshi, Haruki Kume, Yasuhiko Igawa

**Affiliations:** 1 Department of Urology, The Graduate School of Medicine, The University of Tokyo, Tokyo, Japan; 2 Department of Continence Medicine, The Graduate School of Medicine, The University of Tokyo, Tokyo, Japan; 3 Department of Dermatology, The Graduate school of Medicine, The University of Tokyo, Tokyo, Japan; 4 Department of Oral-maxillofacial surgery, The Graduate school of Medicine, The University of Tokyo, Tokyo, Japan

**Keywords:** Vulvar paget’s disease, Urethral stricture, Buccal mucosa

## Abstract

**Background:**

Vulvar Paget’s disease (VPD) is a rare malignant disorder originating in the external genitalia. It occasionally invades into urethral or vaginal mucosa of female, making surgical treatment more complicating. In case of urethral invasion of Paget’s cells, systematic mapping biopsy of urethral mucosa is the standard of care to determine the range of surgical resection. Resection of urethral mucosa and simple skin grafting often result in urethral stricture after surgery, which severely deteriorates patient’s quality of life.

**Case presentation:**

We applied a new technique of advancement urethral meatoplasty using buccal mucosa, in two Japanese cases of VPD with urethral invasion. After broad resection of vulvar skin together with the urethral mucosa, buccal mucosa was implanted between advanced urethral mucosa and skin graft. In both cases, we could prevent urethral stricture one year and two years after surgery, respectively.

**Conclusion:**

This technique prevented urethral stricture after surgery and could be a useful technique as part of urethroplasty for VPD.

## Introduction

Vulvar Paget’s disease (VPD) is a rare malignant skin disease which originates in vulvar apocrine gland bearing cells. It is also called extramammary Paget’s disease, and often manifests as adjacent primary anal, rectal, or bladder adenocarcinoma. Although the true incidence is unclear, the median age at diagnosis is 72, and women have higher occurrence than men [1]. The early symptoms include perineal or vulvar irritation, itching, and pain. The lesion appears as eczematous, papillomatous, or ulcerating erythema. It could sometimes be misdiagnosed as eczema or mycosis. Therefore, early histological diagnosis with skin biopsy is mandatory [1]. 

Surgical resection is the standard of care for VPD. To prevent the recurrence, surgical resection with clear margin is crucial. Especially in case of urethral invasion, adequate excision without unnecessary sacrifice of tissue is important to preserve lower urinary tract function after surgery.

**Fig. 1:**
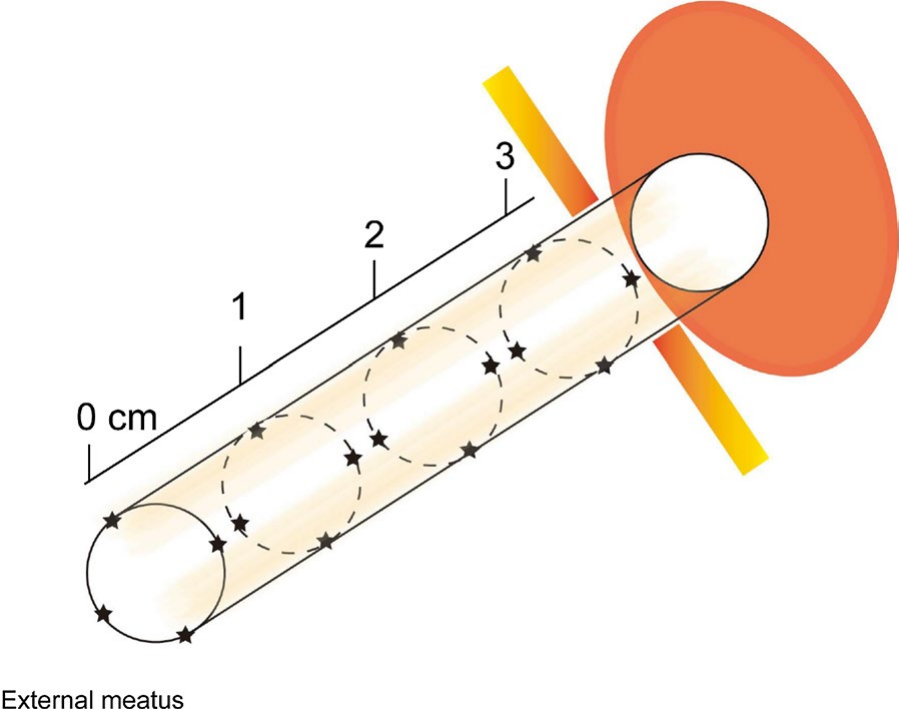
Schema of systematic mapping biopsy of urethral mucosa.

Urethral invasion of VPD is even more infrequent. To determine the length of urethra to resect, or to determine whether or not the urethra could be spared, mapping biopsy of the urethra is quite helpful [2]. Our strategy of mapping biopsy of urethral mucosa is shown in Fig. 1. If the involvement of Paget's cells is limited in the distal urethral mucosa, urinary continence function can be preserved by sparing of the mid- and proximal-urethra. Otherwise, total urethrectomy and urinary diversion is necessary [3].

Disappointingly, however, some of the sphincter-spared patients experience urethral stricture, or urinary scattering at least, after resection of the urethra [4–6]. Development of surgical technique to prevent urethral stricture is our urgent challenge to improve patients’ quality of life.

We present a novel operative technique of advancement urethral meatoplasty utilizing buccal mucosa for Vulvar Paget’s disease that prevented urethral stricture after surgery.

## Case 1

**Fig. 2:**
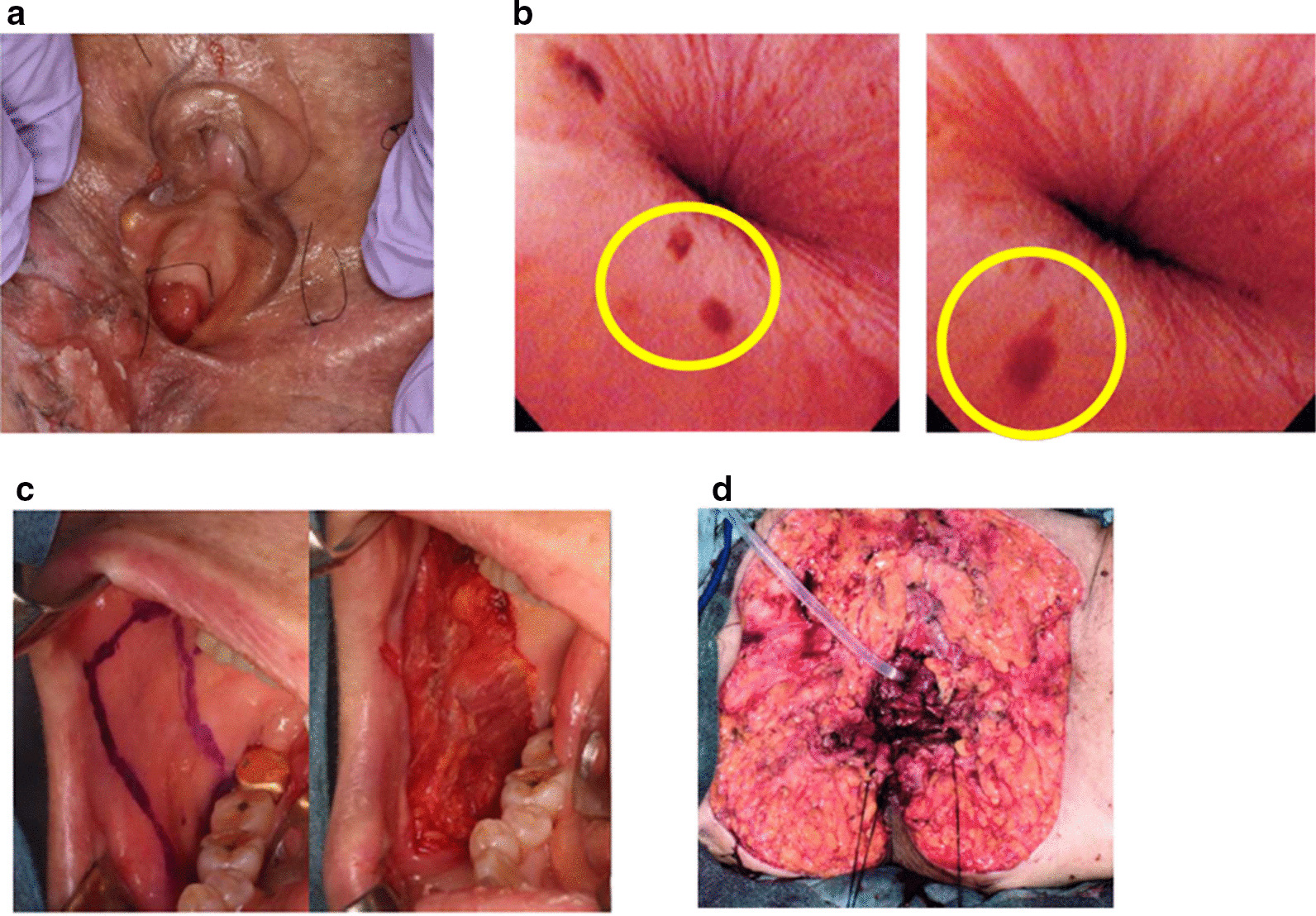
Case 1. **a** Appearance of the external genitalia. **b** Brown spots at the external urethral meatus. Yellow circles indicate brown spots. **c** Pictures of harvesting buccal mucosa. **d** Implantation of buccal mucosa around the advanced urethral mucosa.

An 87-year old Japanese female presented with erythema and papules on her external genitalia, after diagnosis of VPD at another hospital. She underwent broad mapping biopsy at the Department of Dermatology and Paget’s cells were detected at vaginal opening and external urethral meatus (Fig. 2a). She was referred to us, and systematic mapping biopsy of the urethral mucosa was performed under anesthesia. Brown spots were observed on the urethral mucosa by urethroscopy examination (Fig. 2b). Mapping biopsy as shown in Fig. 1 was performed, and it revealed Paget’s cells at the external urethral meatus, but the other part of urethra was free of malignant cells. We have resected vulvar skin and vaginal mucosa broadly, together with urethral mucosa to 2 cm from external urethral opening to ensure adequate margin from the biopsy-positive lesion.

The urethral wall was cut at the ventral and dorsal parts (0 and 6 o'clock positions) and inverted with 3-0 vicryl^®^ suturing. Two pieces of buccal mucosa at a size of 25 mm × 20 mm were harvested from the bucca by an oral-maxillofacial surgeon (Fig. 2c). Submucosal tissue and fat were carefully removed with scissors to prepare a thin layer of mucosa, and the buccal mucosa was implanted on the surface of mucosa-denuded, and inverted, urethral wall along the edge of the urethral mucosa (Fig. 2d). Split thickness skin grafting was done using skin of left thigh to cover the defect of vulvar skin. Two years from the surgery, she is free from recurrence of VPD, and voiding function is intact without any signs of urethral stricture or incontinence.

## Case 2

**Fig. 3:**
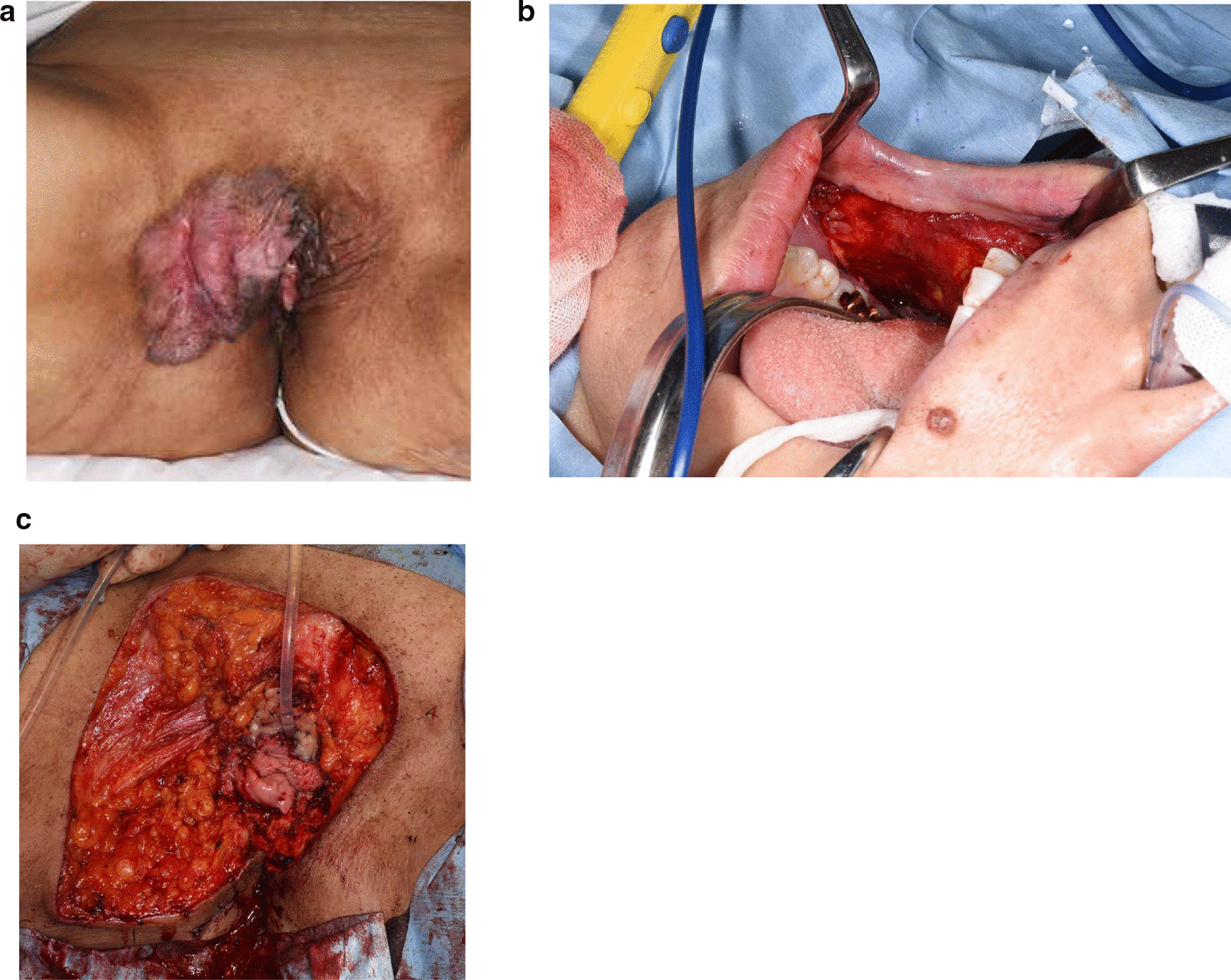
Case 2. **a** Appearance of the external genitalia. **b** Pictures of harvesting buccal mucosa. **c** Implantation of buccal mucosa around the advanced urethral mucosa.

A 59-years old Japanese female with a symptom of itching at extragenital area for 20 years was referred to our hospital. Clinical examination revealed palm sized erythematous lesion with ulcer at vulva and bilateral inguinal lymphadenopathy (Fig. 3a). She was diagnosed with VPD by skin biopsy, and Paget’s cells were present at the external opening of urethra. Broad resection of vulvar skin and urethral mucosa was performed. Initially, urethral mucosa 1 cm from the opening was resected. Rapid frozen pathological test revealed Paget’s cells on the right side of urethral mucosa. We have resected additional 7 mm of mucosa all around and confirmed that the surgical margin was free of Paget’s cells. The urethral wall was cut at the ventral and the dorsal parts and inverted with 3-0 vicryl^®^ suturing. Preparation and implantation of buccal mucosa was done as in Case 1 (Fig. 3b and c). Split thickness skin grafting was done using skin of left thigh to cover the defect of vulvar skin. A year later, no recurrence of VPD were detected. Lower urinary tract function is completely preserved as before operation, and she is now free from any signs of urethral stricture or incontinence.

## Discussion

VPD is a rare intraepithelial carcinoma of the skin occurring in the anogenital area. As Paget’s disease spreads microscopically throughout the epidermis, the margin of the lesion is sometimes unclear [1]. Therefore, wide range of mapping biopsy is mandatory before operation to secure clear margins. According to the General Rules for Clinical and Pathological Studies on Malignant Neoplasms of the Skin, the Japanese Skin Cancer Society recommends multiple perimeter biopsies 3 cm from the visible border of the lesion [7]. Considering its anatomical nature, it is also important to clarify the invasion of malignant cells into adjacent areas including vaginal wall and urethral mucosa. In case urethral invasion is suspected by biopsy at the external urethral opening, a mapping biopsy of the urethral mucosa under anesthesia is subsequently required (Fig. 1). This is to determine the extent of resection of the urethra; i.e. to determine whether lower urinary tract function can be spared or not. The frozen sections during surgery could substitute this preoperative examination, even though high rate of false-negative results from frozen sections have been reported [8, 9].

One of the complications after urethrectomy is urethral stricture. When skin grafting is applied to cover the defect after broad range of skin resection, direct suturing of graft to the edge of urethral mucosa is one option of closure. We have previously applied this direct suturing to three patients. Unfortunately, clinical interview revealed that all of them experienced spraying on urination.

**Fig. 4:**
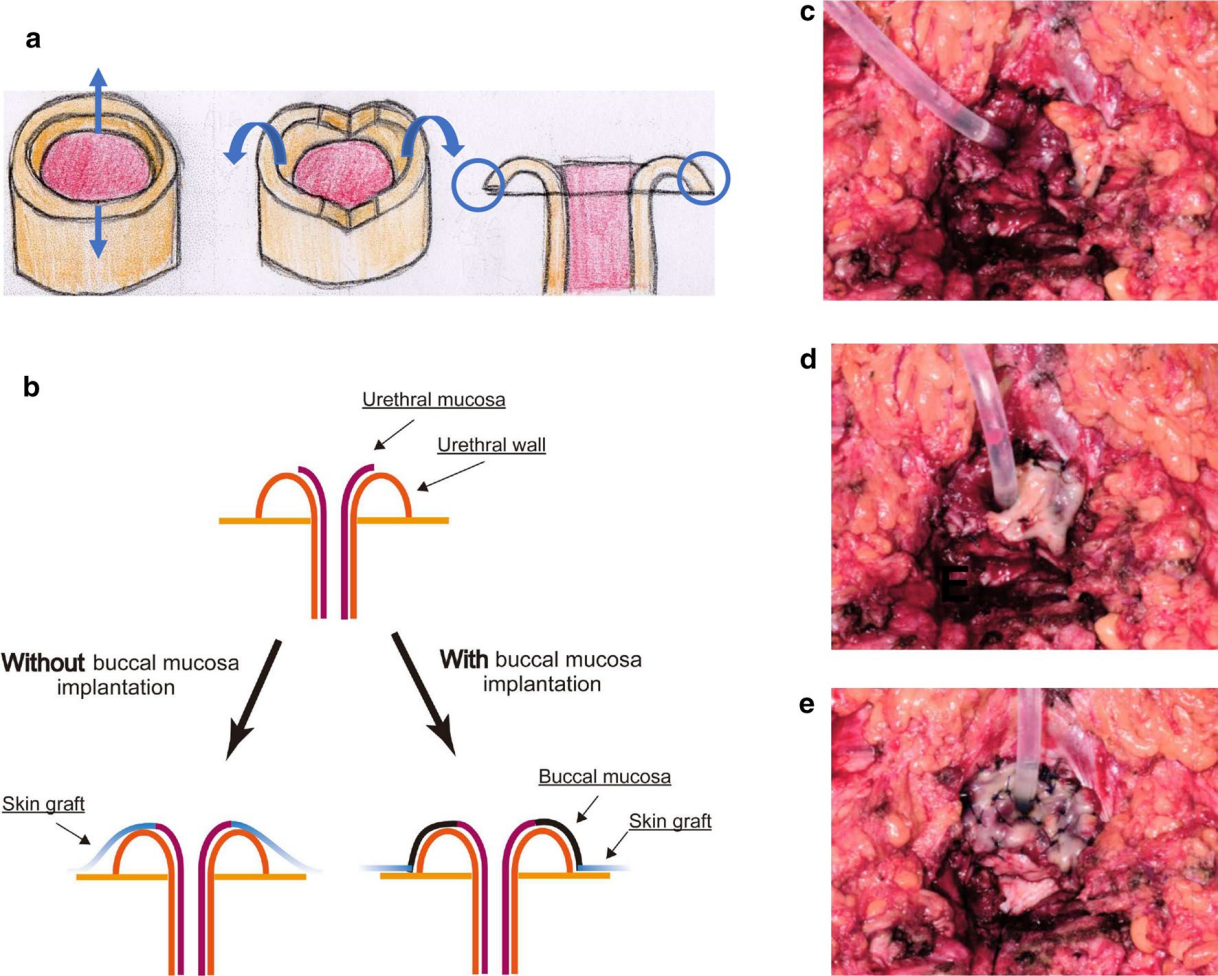
**a** Schema of advancement meatoplasty. **b** Schema of the buccal mucosa implantation between urethral mucosa and skin graft. **c**–**e** Representative pictures of buccal mucosa implantation around advanced urethral mucosa.

**Fig. 5:**
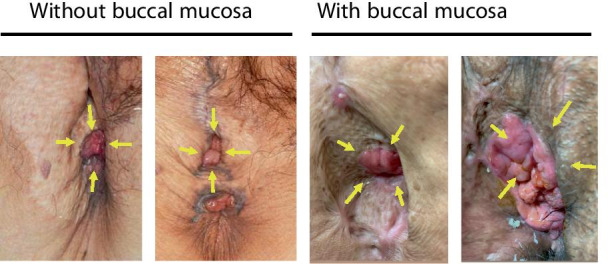
Comparison of appearance of postoperative external urethral meatus. “Without buccal mucosa” (left) and “With buccal mucosa” (right). Pictures from two independent cases each. Yellow arrows indicate external urethral meatus

To prevent the urethral stricture, we have developed a new technique of urethroplasty using buccal mucosa. Historically, there are several techniques of urethroplasty reported. For instance, vaginal flap urethroplasty, labial flap urethroplasty, and vaginal and labial graft urethroplasty have been the popular techniques [4, 10, 11]. Obviously, however, these techniques are not applicable for VPD patients with vaginal and urethral invasion, where vaginal wall and labia are resected simultaneously. For very distal urethral strictures (within 0.5-1.0 cm of external meatus), primary excision and advancement meatoplasty can be applied [11]. Briefly, interrupted sutures are placed in healthy urethral tissue proximal to the stricture to prevent mucosal retraction. The meatus is then circumferentially incised and the healthy urethral mucosa is advanced and sutured to the vaginal mucosa [3]. Our new technique could be recognized as modification of this advancement meatoplasty. By transplanting prepared buccal mucosa between urethral mucosa and the skin graft, the external urethral meatus would be kept smooth compared to the direct suturing of the skin graft (Fig. 4a–e). This is rather simple, but by utilizing this technique, we can prevent urethral stricture or spraying on urination after operation. The comparison of postoperative pictures of VPD patients shows narrow external meatus in the “Without buccal mucosa” group (Fig. 5). Importantly, those two patients in “With buccal mucosa” group do not experience urethral stricture or spraying on urination one year after surgery.

## Conclusion

We report a new technique of advancement urethral meatoplasty using buccal mucosa. This maneuver could be applied in cases of VPD with urethral invasion where valvar skin is broadly resected, and the original structure is damaged. Accumulation of cases and longer follow up data are needed to prove the real utility of this technique.

## Data Availability

Not applicable.

## Abbreviations

VPD: Vulvar Paget’s disease
VPD: Vulvar Paget’s disease
